# Factors associated with missed assessments in a 2-year longitudinal study of acute respiratory distress syndrome survivors

**DOI:** 10.1186/s12874-018-0508-8

**Published:** 2018-06-15

**Authors:** Sara E. Heins, Amy W. Wozniak, Elizabeth Colantuoni, Kristin A. Sepulveda, Pedro A. Mendez-Tellez, Cheryl Dennison-Himmelfarb, Dale M. Needham, Victor D. Dinglas

**Affiliations:** 1 Department of Health Policy and Management, Johns Hopkins Bloomberg School of Public Health, Baltimore, MD USA; 2 Department of Biostatistics, Johns Hopkins Bloomberg School of Public Health, Baltimore, MD USA; 3 Outcomes After Critical Illness and Surgery Group, Division of Pulmonary and Critical Care Medicine, Johns Hopkins University School of Medicine, Baltimore, MD USA; 4 Division of Pulmonary and Critical Care Medicine, School of Medicine, Johns Hopkins University, 1830 E. Monument Street, 5th floor, Baltimore, MD 21287 USA; 5 Department of Anesthesiology and Critical Care Medicine, School of Medicine, Johns Hopkins University, Baltimore, MD USA; 6 Johns Hopkins University School of Nursing, Johns Hopkins University, Baltimore, MD USA; 7 Johns Hopkins Institute for Clinical and Translational Sciences, Johns Hopkins University, Baltimore, MD USA; 8 Department of Physical Medicine and Rehabilitation, School of Medicine, Johns Hopkins University, Baltimore, MD USA

**Keywords:** Acute respiratory distress syndrome, Intensive care, Data quality, Patient outcomes assessments, Prospective studies, Follow-up studies

## Abstract

**Background:**

To evaluate participant-related variables associated with missing assessment(s) at follow-up visits during a longitudinal research study.

**Methods:**

This is a prospective, longitudinal, multi-site study of 196 acute respiratory distress syndrome (ARDS) survivors. More than 30 relevant sociodemographic, physical status, and mental health variables (representing participant characteristics prior to ARDS, at hospital discharge, and at the immediately preceding follow-up visit) were evaluated for association with missed assessments at 3, 6, 12, and 24-month follow-up visits (89–95% retention rates), using binomial logistic regression.

**Results:**

Most participants were male (56%), white (58%), and ≤ high school education (64%). Sociodemographic characteristics were not associated with missed assessments at the initial 3-month visit or subsequent visits. The number of dependencies in Activities of Daily Living (ADLs) at hospital discharge was associated with higher odds of missed assessments at the initial visit (OR: 1.26, 95% CI: 1.12, 1.43). At subsequent 6-, 12-, and 24 months visits, post-hospital discharge physical and psychological status were not associated with subsequent missed assessments. Instead, the following were associated with lower odds of missed assessments: indicators of poorer health prior to hospital admission (inability to walk 5 min (OR: 0.46; 0.23, 0.91), unemployment due to health (OR: 0.47; 0.23, 0.96), and alcohol abuse (OR: 0.53; 0.28, 0.97)) and having the preceding visit at the research clinic rather than at home/facility, or by phone/mail (OR: 0.54; 0.31, 0.96). Inversely, variables associated with higher odds of missed assessments at subsequent visits include: functional dependency prior to hospital admission (i.e. dependency with > = 2 Instrumental Activities of Daily Living (IADLs) (OR: 1.96; 1.08, 3.52), and missing assessments at preceding visit (OR: 2.26; 1.35, 3.79).

**Conclusions:**

During the recovery process after hospital discharge, dependencies in physical functioning (e.g. ADLs, IADLs) prior to hospitalization and at hospital discharge were associated with higher odds of missed assessments. Conversely, other indicators of poorer health at baseline were associated with lower odds of missed assessments after the initial post-discharge visit. To reduce missing assessments, longitudinal clinical research studies may benefit from focusing additional resources on participants with dependencies in physical functioning prior to hospitalization and at hospital discharge.

**Electronic supplementary material:**

The online version of this article (10.1186/s12874-018-0508-8) contains supplementary material, which is available to authorized users.

## Background

There has been increasing interest in evaluating and reporting outcomes after hospital discharge in survivors of critical illness, including in clinical trials in this study population [1]. However, missing data are common in such studies. For example, a review of randomized trials published over a 6-month period in four high impact general medicine journals showed that some primary outcome data was missing for 89% of studies (*n* = 71), and that 18% of studies had missingness rates of more than 20% [2]. High rates of missing data detrimentally impacts statistical power and may introduce selection bias and loss of study validity [3, 4].

Loss to follow-up contributes to missing data, and many studies have examined factors associated with loss to follow-up to identify factors that could reduce attrition and the potential impact of attrition on study findings [5–9]. In addition, patients who are not lost to follow-up, but have missing data from incomplete study visits, also contribute to decreased precision and statistical power and potential selection bias. However, variables associated with missing data beyond loss to follow-up have not been well-studied. Understanding these can assist investigators in anticipating and tailoring follow-up efforts to minimize missing data in participants who attend their follow-up visits.

Survivors of acute respiratory distress syndrome (ARDS) may be especially vulnerable to incomplete follow-up visits during longitudinal studies. Many of these patients have poor baseline health and quality of life [10–13], and often face new or worsened physical and psychological morbidities after hospitalization [14–18]. These impairments may present difficulties for survivors to participate in longitudinal studies. In addition, follow-up research assessments of these individuals tend to be lengthy involving multiple psychological and physical surveys and performance-based tests [19–24]. Hence, our objective is to use data from a multi-site study of ARDS survivors to evaluate patient-related variables associated with missed assessments during follow-up visits over the course of 2-years of longitudinal follow-up.

## Methods

### Study population and design

Mechanically ventilated patients, meeting the American-European Consensus Conference criteria for acute lung injury (ALI) that were in effect during the time of enrollment [25], were enrolled from 13 intensive care units from 4 teaching hospitals in Baltimore, MD (October 2004 – October 2007) [19]. Hereafter, we use the term ARDS, rather than ALI, to be consistent with the more recent Berlin definition [26]. Exclusion criteria included having > 96 h between ARDS diagnosis and enrollment, > 5 days mechanical ventilation before enrollment, pre-existing ARDS when transferred to a study ICU, pre-existing illness with a life expectancy of less than 6 months, any limitation of care at the time of enrollment (e.g. no cardiopulmonary resuscitation status), previous lung resection, inability to speak or understand English, and no fixed address. Prior to hospital discharge, study participants or proxies were administered a retrospective questionnaire on pre-hospitalization health status. Additionally, at hospital discharge, participants were assessed for independence in activities of daily living (ADLs, includes continence, toileting, and feeding), select health symptoms (e.g. shortness of breath), and discharge disposition including any health services required if discharged to home. Lastly, participants completed a battery of patient-reported and performance-based assessments (assessments listed under “Primary Outcome” section) of their physical and psychological status at 3, 6, 12, and 24 months after ARDS.

Follow-up patients from all 4 sites was conducted centrally by the coordinating center (Johns Hopkins University). The research staff collecting follow-up data underwent rigorous training and on-going quality assurance evaluations for conducting all participant assessments. Loss to follow-up in this cohort was minimized using published retention methods [27–30]. Retention strategies included: sending participants letter and magnet with study name/logo and phone number; reminder phone calls and letters for upcoming visits; meal vouchers, free parking or taxi rides; home visits to those unable to come to research clinic; thank you letters after visit; and newsletters and birthday cards to maintain contact between visits [19]. We also offered flexible visit hours (e.g. early or late in the day, and weekend) and home visits.

### Primary outcome

At each of the 3, 6, 12 and 24 month follow-up visits, there were 15 participant assessments of physical and psychological status: 1) Activities of Daily Living (ADLs), 2) Instrumental Activities of Daily Living (IADLs, activities that require more complex thought, e.g. using telephone, managing finances) [31], 3) Hearing Handicap Inventory for Adults-Screening (HHIA-S) [32], 4) EQ-5D [33], 5) Short-Form 36 Questionnaire v2 (SF-36) [34], 6) Hospital Anxiety and Depression Scale (HADS) [35], 7) Impact of Event Scale-Revised (IES-R) [36], 8) 6-min walk distance [37], 9) manual muscle testing (MMT) [38], 10) hand grip strength [39], 11) maximal inspiratory pressure (MIP) [40], 12) Telephone Interview of Cognitive Status (TICS) [41], 13) Sydney Swallowing Questionnaire (SSQ) [42], 14) anthropometric measurements, and 15) a collection of miscellaneous questions about employment, caregiver, etc. There were a small number of assessments that were not applicable to some participants (e.g. contraindications, comatose/cognitive status, amputated limbs or digits), and the number of possible assessments were reduced from maximum of 15. For the purposes of this analysis, assessments that were missed for reasons unrelated to participant factors (e.g. staff or equipment unavailable to conduct assessment) were considered “not applicable” and the total number of possible assessments was modified. Partially completed assessments (i.e. individual surveys or tests) were not considered missed. Reasons for missed or incomplete visits were categorized as due to the physical status of the participant (poor physical condition cited as the reason for not completing the assessment, although no explicit contraindication was present), refusal, lost contact, and other.

The outcome of interest was the number of missed assessments out of the number of possible assessments at each follow-up visit. Participants who missed an entire visit are included in analyses and considered to have missed 100% of the assessments at that visit.

### Variables evaluated for association with missed assessments

Several baseline and pre-hospitalization variables were considered including participant demographics (age, sex, race, and education level), unemployment due to health condition, whether or not the participant resided at home without healthcare services, inability to walk at least 5 min, Charlson Comorbidity Index (CCI) [43], Functional Comorbidity Index (FCI) [44], and retrospectively collected baseline ADLs, IADLs, EQ-5D and SF-36. History of excessive alcohol use, illicit drug use, and any psychiatric comorbidity were collected from medical record. At hospital discharge, patients were evaluated for shortness of breath, ADLs, and discharge location. At each follow-up visit the following variables were evaluated: ADLs, IADLs, shortness of breath, participant living location, HHIA-S score, unemployment due to health, EQ-5D Visual Analogue Scale (VAS) (range: 0 to 100; higher score is better) and utility scores (range: − 0.11 to 1.0; higher score is better), SF-36 Physical Component Score (PCS) and Mental Component Score (MCS) (mean of 50, SD = 10; higher score is better), HADS anxiety and depression subscales scores (for each, range: 0 to 21; lower score is better, with scores ≥8 indicating substantial symptoms), IES-R score (range: 0 to 4; lower score is better ≥1.6 indicating substantial symptoms), 6-min walk test (percent of predicted value), MMT strength (score range: 0 to 60; higher score is better), hand grip strength (percent of predicted value), MIP (percent of predicted value), missing at least 1 assessment, and whether all data was collected at the research clinic (vs. via phone or mail, or visit to the participant’s home or long-term care facility). For all time-points, ADLs variable was defined as number of ADLs dependencies (out of a possible six activities) or dichotomized as ≥1 vs. 0 ADL dependencies. Similarly, the IADLs variable was defined as number of IADLs dependencies (out of a possible eight activities) or dichotomized as ≥2 vs. < 2 IADLs dependencies.

### Analysis

For all patients at all follow-ups, we assumed that the outcome, the number of missed assessments, followed a Binomial distribution with required parameters: the total number of possible assessments and the mean, the probability of a missed assessment. Assuming the outcome follows the Binomial distribution implies that the probability of a missed assessment is the same for all possible assessments for the patient at the given follow-up. To quantify the association between the probability of a missed assessment (i.e. the mean of the outcome variable) with the a priori identified exposure variables, Binomial logistic regression models were used that accounted for variation in the total number of possible assessments across patients and follow-ups [45, 46]. More details on the Binomial logistic regression model can be found in the Additional file 1. In the Binomial logistic regression model, associations were quantified using odds ratio, i.e. the relative odds of a missed assessment per unit change in the exposure variable of interest. Standard errors for the odds ratios were estimated using robust variance estimates to account for the potential over- or under-dispersion in the assumed Binomial variance. First, pre-ARDS baseline and hospital discharge variables were correlated with the number of missed assessments at the initial follow-up at 3-months via bivariable Binomial regression models. For evaluating missed assessments across 6-, 12-, and 24-months, longitudinal Binomial logistic regression models fit with generalized estimating equations with an exchangeable correlation structure were used. The longitudinal models included main effects for follow-up time, exposure and their interaction. For variables where the relationship did not vary over time, the interaction term was dropped from subsequent models. Exposure variables were selected for inclusion in final multivariable models based on *p* < 0.20 for their univariable association with the outcome. If two definitions of the same variable were significant at p < 0.20 (for example, ≥1 ADL and number of ADLs), the one with the stronger association (i.e., smaller *p*-value) was used in the multivariable model.

There was minimal missing data for baseline and discharge characteristics. However, in the longitudinal models, exposures measured at the prior visit were included (i.e. 3-month IADLs as an exposure for missed assessments at 6-months) and could contain missing data. In these models, an indicator for whether or not the exposure was assessed at the prior visit was included as well as the interaction between this indicator and the exposure. Linearity of the association of each continuous exposure variable was assessed using locally weighted scatterplot smoothing (LOWESS) and restricted cubic splines, and there were no continuous exposures for which the linearity assumption was strongly violated. Standard regression diagnostics were used to assess model fit (evaluated by comparing predicted versus observed values and comparing quasi-information criteria (QIC) between multivariable models), influential data points (evaluated by Cooks D), and multicollinearity (evaluated by Variance Inflation Factors (VIFS)) (See Additional file 1: Table S2 for results). Logit of proportion of missed assessments was calculated for each person at each time-point in order to visualize the associations between the outcome variable and significant variables. Logit of proportion of missed assessments was undefined when probability was 0 or 1; therefore these logits were set to − 4 and 4, respectively, for the purpose of these illustrations. Figures were then created by calculating the univariable means of the logit of proportion of missed assessment with corresponding 95% confidence intervals or regression line, where appropriate.

A two-sided *p*-value < 0.05 was used to indicate statistical significance in the final multivariable models. All statistical analyses were performed using SAS version 9.3.

## Results

**Fig. 1:**
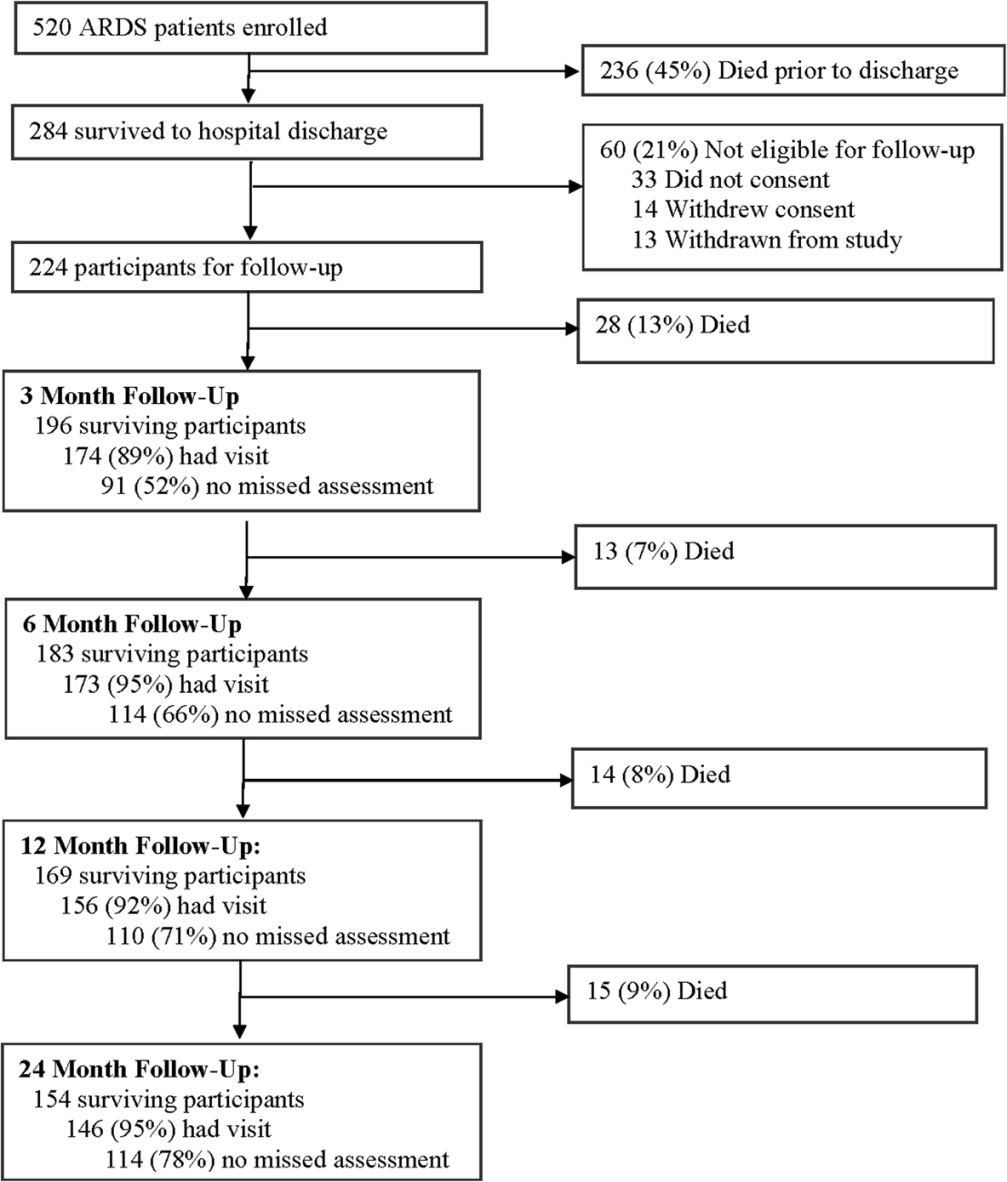
Flow Diagram of Study Participants

The study population was comprised of 196 participants who survived to 3-months and consented for 2-year longitudinal follow-up (Fig. 1). The majority of participants were male (56%), white (58%), and had no more than high school education (64%) (Table 1). Some participants had a history of alcohol abuse (25%), drug abuse (33%), or other psychiatric comorbidity (27%) prior to hospitalization. During follow-up, survivors generally experienced some improvement in health status (Table 2); for instance, the proportion of participants living at home without services increased from 63% at 3-months to 85% at 24-months. Only a small proportion of participants had completely missed visits (ranging from 11% at 3-month visit to 5% at 6- and 24-month visit), but incomplete visits (i.e. missing at least 1 of 15 assessments during the comprehensive visit) were more common (ranging from 48% at 3-month visit to 22% at 24-month visit) (Fig. 1, see Additional file 1: Table S1 for summary of missed assessment by outcome).

**Table 1:** Participant characteristics for those alive at 3-month follow-up

Baseline Characteristics^a^	*n* = 196
Age (years)	49 (40, 58)
Male, No. (%)	110 (56)
White race, No. (%)	113 (58)
No more than high school education, No. (%)	117 (64)
Charlson Comorbidity Index	1 (0, 3)
Functional Comorbidity Index	1 (1, 3)
Alcohol abuse, No. (%)	49 (25)
Drug abuse, No. (%)	65 (33)
Psychiatric comorbidity (any), No. (%)	52 (27)
Unable to walk for at least 5 min, No. (%)	38 (20)
Residing at home without services, No. (%)	177 (91)
Unemployed due to health, No. (%)	57 (30)
Number of dependent ADLs	0 (0, 0)
Dependent in any ADLs, No. (%)	31 (16)
Number of dependent IADLs	1 (0, 3)
Dependent in ≥ 2 IADLs, No. (%)	76 (40)
EQ-5D Visual Analogue Scale (range: 0 to 100)	75 (50, 90)
EQ-5D utility score (range − 0.11 to 1.0)	0.8 (0.5, 1)
SF-36 PCS (mean = 50, SD = 10)	45 (35, 55)
SF-36 MCS (mean = 50, SD = 10)	49 (37, 57)
Status at hospital discharge	
Discharged to home without services, No. (%)	46 (24)
Number of dependent ADLs	4 (1, 6)
Dependent in any ADLs, No. (%)	147 (75)
Shortness of breath, No. (%)	45 (28)

Abbreviations: *ADL* Activities of daily living, *EQ-5D* EuroQOL-5D, *VAS* Visual analog scale, *IADL* instrumental activities of daily living, *SF-36* Short-form 36 functional assessment, *MCS* mental component score, *PCS* Physical component score

^a^Data are presented as median (interquartile range), unless stated otherwise. Missing data: Education, 12; Unable to perform 5 min walk, 4; Patient location, 2; Baseline employment, 5; ADL, 3; IADL, 6; VAS, 44; Utility, 38; SF-36 PCS, 42; SF-36 MCS, 42; Discharge location 1, Discharge ADLs 1, Discharge shortness of breath, 38

**Table 2:** Participant characteristics and outcomes summaries for those alive at follow-ups

Follow-up Variables^a^	3-Months (*n* = 174)	6-Months (*n* = 173)	12-Months (*n* = 156)	24-Months (*n* = 146)
Living at home without services, No. (%)	106 (63)	129 (75)	123 (80)	122 (85)
Number of dependent ADLs	0 (0, 1)	0 (0, 0)	0 (0, 0)	0 (0, 0)
Dependent in any ADLs, No. (%)	55 (33)	40 (23)	35 (23)	30 (21)
Number of dependent IADLs	3 (0, 6)	2 (0, 5)	1 (0, 4)	1 (0, 4)
Dependent in ≥2 IADLs, No. (%)	111 (66)	93 (54)	68 (44)	66 (46)
Shortness of breath, No. (%)	57 (36)	59 (36)	53 (36)	49 (35)
HHIA-S Score	0 (0, 4)	0 (0, 2)	0 (0, 4)	0 (0, 8)
Unemployed due to health, No. (%)	91 (54)	88 (51)	81 (53)	68 (47)
EQ-5D Visual Analogue Scale	70 (50, 80)	70 (50, 80)	70 (50, 83)	70 (50, 90)
EQ-5D utility score	0.76 (0.40, 0.83)	0.76 (0.47, 0.84)	0.78 (0.60, 0.84)	0.79 (0.58, 0.84)
SF-36 PCS	34 (28, 43)	36 (28, 46)	41 (31, 49)	41 (34, 51)
SF-36 MCS	48 (35, 56)	50 (39, 59)	50 (37, 59)	49 (38, 57)
HADS Anxiety Score	6 (3, 9)	5 (2, 9)	6 (2, 9)	6 (2, 10)
HADS Anxiety Score ≥ 8, No. (%)	58 (38)	52 (32)	50 (35)	55 (40)
HADS Depression Score	5 (3, 8)	5 (2, 8)	4 (2, 7)	4 (1, 9)
HADS Depression Score ≥ 8, No. (%)	40 (26)	41 (25)	34 (24)	44 (32)
IES-R Total Score	0.9 (0.4, 1.6)	0.7 (0.1, 1.4)	0.6 (0.2, 1.5)	0.7 (0.2, 1.5)
IES-R Total Score ≥ 1.6, No. (%)	36 (24)	31 (19)	31 (22)	31 (23)
6-min Walk Test, % Predicted	51 (31, 66)	58 (42, 71)	63 (45, 73)	66 (42, 81)
MMT strength score	53 (49, 58)	54 (50, 58)	56 (52, 59)	57 (52, 60)
Grip, % predicted	65 (51, 85)	73 (60, 91)	80 (64, 100)	83 (67, 99)
MIP, % predicted	74 (59, 93)	75 (56, 103)	85 (61, 110)	95 (64, 118)

Abbreviations: *ADL* Activities of daily living, *EQ-5D* European quality of life index, *IADL* instrumental activities of daily living, *IES-R* Impact of event scale-revised, *HADS* Hospital anxiety and depression score, *HHIA-S* Hearing handicap inventory for adults-screening, *IES-R* Impact of event scale-revised, *ICU* Intensive care unit, *LOS* Length of stay, *SF-36* Short-form 36 functional assessment, *MCS* Mental component score, *MIP* Maximal inspiratory pressure, *MMT* Manual muscle testing, *PCS* Physical component score, *VAS* Visual analog scale

^a^Data are presented as median (interquartile range), unless stated otherwise. Missing data at 3, 6, 12, and 24 months, respectively (includes missing due to contraindication, unrelated to participant factors, and partially completed assessments that could not be scored): Patient Location 5, 1, 2, 2; ADLs 5, 1, 1, 2; IADLS 5, 1, 1, 2; Shortness of Breath 14, 8, 8, 5; HHIA-S 20, 13, 14, 12; Unemployment 5, 1, 2, 2; EQ-5D 15, 8, 8, 9; SF-36 20, 9, 10, 9; HADS 22, 12, 14, 10; IES-R 23, 13, 15, 11; 6-Minute Walk 61, 49, 37, 31; MMT 42, 31, 26, 16; Grip 46, 33, 27, 17; MIP 59, 44, 34, 19

At 3-month follow-up, 105 (54%) of 196 participants missed at least 1 of the possible assessments. The median (interquartile range [IQR]) total number of possible assessments was 15 (15, 15) with a median (IQR) percent of missed assessments 7% (0, 33%). The most common reason for missing assessments was participant’s physical status (e.g. hospitalized, illness, fatigued) (46% of visits), followed by other reasons (e.g. incarcerated, lives too far, lacks time) (23%), refusal (18%), and lost contact with participant (13%). Of the 21 a priori variables evaluated for association with missed assessments at 3-month follow-up, 4 were included in the multivariable model (Table 3). Only dependencies in ADLs at hospital discharge (odds ratio (OR) of 1.26 [95% Confidence Interval [CI]: 1.12, 1.43; p = < 0.001] per 1 additional dependency) was independently associated with missed assessments at 3-month visit. Plots to visualize these associations are available in Additional file 1: Figure S1.

**Table 3:** Factors associated with missed assessments at the initial visit at 3-months after ARDS

	Univariable OR^a^ (95% CI)	P-Value^a^	Multivariable OR^a^ (95% CI)	P-Value^a^
Age	1.01 (0.99, 1.02)	0.511		
Male	0.76 (0.44, 1.33)	0.336		
White race	0.66 (0.38, 1.15)	0.144	0.95 (0.48, 1.87)	0.874
No more than high school education	1.74 (0.96, 3.17)	0.070	0.93 (0.50, 1.70)	0.806
Comorbidity				
Charlson Comorbidity Index	1.06 (0.96, 1.19)	0.256		
Functional Comorbidity Index	1.06 (0.88, 1.28)	0.553		
Alcohol abuse	0.87 (0.45, 1.67)	0.681		
Drug abuse	0.85 (0.47, 1.54)	0.586		
Psychiatric comorbidity	1.62 (0.88, 2.99)	0.123	1.28 (0.56, 2.92)	0.561
Baseline status prior to ARDS				
Unable to walk for at least 5 min	0.90 (0.46, 1.79)	0.772		
Residing at home without services	0.67 (0.26, 1.70)	0.394		
Unemployed due to health	1.33 (0.74, 2.40)	0.338		
Number of dependent ADLs^b^	0.93 (0.73, 1.18)	0.545		
Dependent in any ADLs^b^	0.98 (0.45, 2.13)	0.968		
Number of dependent IADLs^b^	1.01 (0.90, 1.13)	0.879		
Dependent in ≥2 IADLs^b^	1.11 (0.63, 1.97)	0.723		
EQ-5D Visual Analogue Scale	1.00 (0.99, 1.01)	0.977		
EQ-5D Utility score	1.65 (0.48, 5.65)	0.425		
SF-36 PCS	1.00 (0.97, 1.02)	0.893		
SF-36 MCS	1.01 (0.99, 1.04)	0.211		
At hospital discharge				
Discharged to home without services	0.79 (0.40, 1.58)	0.513		
Number of dependent ADLs^b^	1.10 (0.97, 1.24)	0.125	1.26 (1.12, 1.43)	< 0.001
Dependent in any ADLs^b^	1.75 (0.85, 3.61)	0.132		
Shortness of breath	1.06 (0.56, 2.03)	0.853		

Abbreviations: *ADL* Activities of daily living, *ARDS* Acute respiratory distress syndrome, *EQ-5D* EuroQOL-5D, *IADL* Instrumental activities of daily living, *ICU* Intensive care unit, *MCS* Mental component score, *PCS* Physical component score, *SF-36* Short-form 36 functional assessment

^a^Estimates and *P*-values were calculated with binomial regression model using robust variance estimates. Variables significant at the *p* < 0.20 level were selected for the multivariable model. Multivariable models included all factors presented in the column

^b^If two definitions of the same variable had *p* < 0.20 (example: > = 1 ADL and No. of ADLS), the one with the stronger association (i.e. lowest *p*-value) was used in the multivariable model

Between 6 and 24 month follow-up, 103 (56%) of 183 participants had at least one missing assessment. The median (IQR) total number of possible assessments was 15 (15, 15), 15 (15, 15) and 15 (15, 15) for the 6, 12, and 24 month follow-up, respectively. The median percent of missed assessments was relatively stable over time; 0% (0, 13%), 0% (0, 7%) and 0% (0, 7%) at 6, 12, and 24 month follow-up, respectively. The most common reason for a missing assessment was participant physical status (46% of visits), refusal (24%), other reasons (24%), and lost contact with participant (5%). Of the 37 a priori variables evaluated for association with missed assessments at 6-, 12-, 24-months, 7 were included in the multivariable model (Table 4). One variable, IES-R score ≥ 1.6, over time had significantly different associations with missed assessments at subsequent visit. However, when this variable and interaction term (with time) were added to the multivariable model, results remained consistent and goodness of fit decreased. Therefore, this variable was excluded from the multivariable model. Based on the final multivariable model evaluating missed assessments over 6–24 month follow-up, variables associated with lower odds of missed assessments were: poorer health at baseline: unable to walk 5 min (OR: 0.46; 95% CI: 0.23–0.91), unemployment due to health (0.47; 95% CI:0.23–0.96), and alcohol abuse (OR: 0.53; 95% CI: 0.28–0.97), and prior visit at the research clinic vs. any other location (OR: 0.54; 95% CI: 0.31–0.96). Conversely, variables associated with higher odds of missed assessments were: ≥2 IADL dependencies prior to hospital admission (OR 1.96; 95% CI: 1.08–3.52) and having missed assessments at the prior follow-up (OR 2.26; 95% CI: 1.35–3.79). Plots to visualize these associations are available in Additional file 1: Figure S2.

**Table 4:** Factors associated with missed assessments over 6-, 12, and 24-month follow-up after ARDS^a^

	Univariable OR^a^ (95% CI)	P-Value^a^	Multivariable OR^a^ (95% CI)	P-Value^a^
Age	0.99 (0.97, 1.01)	0.231		
Male	0.80 (0.47, 1.35)	0.406		
Race, White	0.71 (0.42, 1.21)	0.210		
No more than high school education	1.20 (0.69, 2.11)	0.517		
Comorbidity				
Charlson Comorbidity Index	1.02 (0.91, 1.14)	0.715		
Functional Comorbidity Index	0.97 (0.80, 1.17)	0.744		
Alcohol abuse	0.48 (0.25, 0.93)	0.030	0.53 (0.28, 0.97)	0.040
Drug abuse	1.35 (0.76, 2.40)	0.307		
Psychiatric comorbidity	1.43 (0.81, 2.54)	0.217		
Baseline status prior to ARDS				
Unable to walk for at least 5 min	0.46 (0.21, 0.99)	0.048	0.46 (0.23, 0.91)	0.027
Residing at Home without services	1.21 (0.51, 2.87)	0.674		
Unemployed due to health	0.59 (0.32, 1.09)	0.093	0.47 (0.23, 0.96)	0.039
Number of dependent ADLs^c^	1.06 (0.87, 1.28)	0.571		
Dependent in any ADLs^c^	1.55 (0.76, 3.14)	0.226		
Number of dependent IADLs^c^	1.03 (0.94, 1.14)	0.512		
Dependent in ≥2 IADLs^c^	1.49 (0.85, 2.61)	0.162	1.96 (1.08, 3.52)	0.026
EQ-5D-Visual Analogue Scale	1.00 (0.99, 1.01)	0.776		
EQ-5D-Utility score	0.82 (0.24, 2.79)	0.754		
SF-36 Physical Component Score	1.00 (0.98, 1.03)	0.915		
SF-36 Mental Component Score	1.00 (0.97, 1.02)	0.753		
From Prior Follow-up Visit				
Visit Incomplete	2.07 (1.33, 3.23)	0.001	2.26 (1.35, 3.79)	0.002
Entire visit conducted in clinic	0.55 (0.33, 0.91)	0.019	0.54 (0.31, 0.96)	0.035
Living at home without services	1.35 (0.77, 2.37)	0.299		
Number of dependent ADLs^c^	1.04 (0.93, 1.17)	0.507		
Dependent in any ADLs^c^	1.05 (0.63, 1.75)	0.850		
Number of dependent IADLs^c^	0.95 (0.87, 1.04)	0.297		
Dependent in ≥2 IADLs^c^	0.74 (0.47, 1.16)	0.194	0.80 (0.48, 1.33)	0.388
Shortness of breath	1.06 (0.66, 1.72)	0.810		
HHIA-S Score	0.98 (0.95, 1.02)	0.343		
Unemployed due to Health	1.01 (0.62, 1.64)	0.973		
EQ-5D-Visual Analogue Scale	1.00 (0.99, 1.01)	0.441		
EQ-5D-Utility score, per .01 increase	1.00 (0.99, 1.01)	0.772		
SF-36 Physical Component Score	1.01 (0.99, 1.03)	0.341		
SF-36 Mental Component Score	1.00 (0.98, 1.02)	0.887		
HADS Anxiety Score	0.99 (0.95, 1.04)	0.761		
HADS Anxiety Score ≥ 8	1.08 (0.66, 1.76)	0.765		
HADS Depression Score	1.01 (0.95, 1.08)	0.759		
HADS Depression Score ≥ 8	0.91 (0.54, 1.53)	0.721		
IES-R Total Score	0.92 (0.71, 1.20)	0.556		
IES-R Total Score ≥ 1.6^b^	0.76 (0.42, 1.40)	0.385		
6-min Walk Test, % Predicted	1.00 (0.99, 1.01)	0.490		
MMT Score	1.01 (0.97, 1.04)	0.722		
Grip, % Predicted	1.00 (0.99, 1.01)	0.588		
MIP, % Predicted	1.00 (0.99, 1.01)	0.982		

Abbreviations: *ADL* Activities of daily living, *EQ-5D* EuroQOL-5D, *IADL* Instrumental activities of daily living, *IES-R* Impact of event scale-revised, *HADS* Hospital anxiety and depression scale, *HHIA-S* Hearing handicap inventory for adults-screening, *ICU* Intensive care unit, *LOS* Length of stay, *SF-36* Short-form 36, *MCS* Mental component score, *MIP* Maximal inspiratory pressure, *MMT* Manual muscle testing, *PCS* Physical component score, *VAS* Visual analog scale

^a^Estimates and p-values were calculated with binomial regression model using generalized estimating equations with an exchangeable correlation structure. Bivariable models included the exposure presented in each row and indicator variables for time. Variables significant at the p < 0.20 level were selected for the multivariable model. Multivariable models included all factors presented in the column and indicator variables for time. If a model contained data from a prior visit, the model also included a missing data indicator for that variable

^b^Variable had a significantly different association with missed assessments over time. However, when the variable and interactions with time were added to the final multivariable model, results remained consistent. Therefore, these variables are excluded from the multivariable model

^c^If two definitions of the same variables were significant a *p* < 0.20 (example: > = 1 ADL and No. of ADLS), the one with the stronger association (i.e. lower *p*-value) was used in the multivariable model

## Discussion

In this prospective, longitudinal cohort study of 196 ARDS survivors, participant sociodemographic characteristics were not associated with missed assessments at either the initial 3-month visit or subsequent visits at 6-, 12-, and 24-months. ADLs at hospital discharge was associated with higher odds of missed assessments at the initial 3-month follow-up visit. At subsequent visits, post-discharge physical and mental health status were not associated with missed assessments. Instead, baseline (prior to hospitalization) IADLs along with missing assessments at preceding visit were associated with higher odds of missed assessments. Conversely, alcohol abuse and indicators of poor baseline physical health along with completing the preceding visit entirely at the research clinic (vs. other location or mode e.g. home, phone) were independently associated with lower odds of missed assessments at 6-, 12-, and 24-months**.**

To our knowledge, the present study is one of the first to evaluate factors associated with missed assessments within follow-up research visits of ARDS survivors. In our study, no sociodemographic characteristics were associated with missed assessments during follow-up visits. In contrast, studies of cohort attrition have found associations of loss to follow-up with sex, race, and economic status [5, 47–49]. After hospital discharge, health status measures, evaluated via 17 variables in this analysis, were not associated with missed assessments in subsequent visits. In studies of cohort attrition, researchers have found that psychiatric comorbidity was associated with increased odds of loss to follow-up [5, 6, 8]. Our dissimilar findings may be due to the different patient populations studied in these attrition studies or it may be that factors associated with attrition are truly different from factors associated with missing data in those who do attend visits. The latter hypothesis, if correct, highlights that our results complement findings from attrition studies, and that both must be considered to design effective strategies to mitigate missing data.

In the present study, indicators of poor pre-ARDS baseline health (i.e., alcohol abuse, inability to walk for 5 min, and unemployed due to health reason) were independently associated with lower odds of missed assessments. Conversely, dependencies in physical functioning (i.e. ADL and IADL) were associated with higher odds of missed assessments at both the initial visit and at subsequent visits. It is important to note the opposite direction of associations of baseline pre-hospital physical functioning (IADLs) versus other baseline indicators of health with missing assessments. This finding may reflect participants having greater availability (e.g. not working) to participate in research studies, but if their health limitations are severe enough, manifesting as dependencies in IADLs, then they face difficulty in completing the entire battery of assessments during each follow-up research visit. Similar to our findings, a study of burn injury patients, demonstrated higher odds of attrition for those with no pre-existing physical disability [4]. For participants who may have difficulty completing an entire battery of assessments, researchers may choose to prioritize to assess more important outcomes (e.g. primary outcome) ahead of secondary outcomes and to spread out the participants testing over more than one assessment to shorten the duration of each assessment. Notably, in our study, missingness was higher in performance-based measures (i.e. requiring in-person assessments – e.g. 6 min walk test) versus patient-reported outcomes (i.e., surveys that are often simpler and can be done by phone – e.g. EQ-5D). Feasibility of the proposed assessments is one of many issues that researchers should consider in designing their follow-up studies.

This study has a number of strengths, including low levels of participant attrition via extensive use of participant retention strategies as described in the Methods section and extensive collection of baseline demographic information, comorbidity and health status data, along with detailed longitudinal assessments of physical and mental health status for these analyses. Our team was well-trained and adhered to the study’s detailed retention protocol. The longitudinal design allowed us to examine changes in associations over time, with the finding that associations remained relatively constant over time. Despite these strengths, there are potential limitations. First, baseline health, functional, and quality of life status prior to hospital admission were obtained from retrospective interviews, which may introduce recall bias. The inability to obtain prospective baseline status is an inherent challenge in studies involving ARDS patients given the emergent and unpredictable nature of ARDS onset. Second, the cohort retention efforts employed in this study may differ from other studies, affecting generalizability of the associations observed in the factors we evaluated. We did not evaluate the association of study team factors with missing assessments in this study, though factors such as limitations in staff availability and training may have been contributed to missing assessments. However, missing assessments unrelated to participant factors (e.g. staff availability) were excluded from consideration as a “missed” assessment. Finally, the results may not be generalizable to other patient populations as the study involved patients with ARDS (*n* = 196 at first follow-up) from four urban hospitals in one city.

## Conclusions

In conclusion, within the setting of a prospective multisite longitudinal cohort study, we evaluated > 30 variables for associations with missed assessments during follow-up research visits. Baseline sociodemographic characteristics and post-discharge physical and mental health status were not associated with missed assessments during follow-up visits. However, physical functioning prior to study enrollment and at hospital discharge, indicators of poor baseline health and alcohol abuse, and participant history of research visits were each independently associated with missing assessments during follow-up research visits. Investigators planning longitudinal follow-up studies should collect information on baseline health status, physical functioning at hospital discharge, and status of preceding visits to identify participants at risk of missing assessments. This relatively small number of easy to collect data offer invaluable insights for tailoring retention and visit completion efforts to mitigate missing assessments at each follow-up visit.

## Additional file

Additional file 1: “Supplementary Material for Factors Associated with Missed Assessments in a 2-year Longitudinal Study of Acute Respiratory Distress Syndrome Survivors”, This file contains the following: Analysis – Additional explanation of binomial logistic regression, **Table S1.** Number (percentage) of missed assessments over 3-, 6-, 12, and 24-month follow-up after ARDS, **Table S2.** Results of Regression Diagnostics, **Figure S1.** Mean logit of proportion of missed assessment for variables included in the 3-month multivariable model, **Figure S2.** Mean logit of proportion of missed assessment for variables included in the 6–12-24-month multivariable model. (PDF 297 kb)
