# Supplementary material for: Factors associated with missed assessments in a 2-year longitudinal study of acute respiratory distress syndrome survivors
Source: BMC Med Res Methodol. 2018 Jun 15;18:55. doi: 10.1186/s12874-018-0508-8 (PMC6003179; doi:10.1186/s12874-018-0508-8)
Supplement: Supplementary file 1 — “Supplementary Material for Factors Associated with Missed Assessments in a 2-year Longitudinal Study of Acute Respiratory Distress Syndrome Survivors”, This file contains the following: Analysis – Additional explanation of binomial logistic regression, Table S1. Number (percentage) of missed assessments over 3-, 6-, 12, and 24-month follow-up after ARDS, Table S2. Results of Regression Diagnostics, Figure S1. Mean logit of proportion of missed assessment for variables included in the 3-month multivariable model, Figure S2. Mean logit of proportion of missed assessment for variables included in the 6–12-24-month multivariable model. (PDF 297 kb) [file 12874_2018_508_MOESM1_ESM.pdf]

Supplementary Material for

Factors associated with missed assessments in a 2-year longitudinal study of acute respiratory distress syndrome survivors

Sara E. Heins, BA[1]
Amy W. Wozniak, MS[2,3]
Elizabeth Colantuoni, PhD[2,3]
Kristin A. Sepulveda, BA[3,4]
Pedro A. Mendez-Tellez, MD[3,5]
Cheryl Dennison-Himmelfarb, PhD, RN, FAAN [3,6,7]
Dale M. Needham, FCPA, MD, PhD[3,4,8]
Victor D. Dinglas, MPH[3,4]

[1]Department of Health Policy and Management, Johns Hopkins Bloomberg School of Public Health, Baltimore, MD
[2]Department of Biostatistics, Johns Hopkins Bloomberg School of Public Health, Baltimore, MD
[3]Outcomes After Critical Illness and Surgery Group, Division of Pulmonary and Critical Care Medicine, Johns Hopkins University School of Medicine, Baltimore, MD
[4]Division of Pulmonary and Critical Care Medicine, School of Medicine, Johns Hopkins University, Baltimore, MD
[5]Department of Anesthesiology and Critical Care Medicine, School of Medicine, Johns Hopkins University, Baltimore, MD
[6]Johns Hopkins University School of Nursing, Johns Hopkins University, Baltimore, Maryland
[7]Johns Hopkins Institute for Clinical and Translational Sciences, Johns Hopkins University, Baltimore, Maryland
[8]Department of Physical Medicine and Rehabilitation, School of Medicine, Johns Hopkins University, Baltimore, MD

Analysis

Our study measured physical and mental health status in ARDS survivors at 3, 6, 12, and 24-months following ARDS onset. At each follow-up, there were 15 assessments administered to the patient. There were a small number of assessments that were not applicable to some patients (e.g. contraindications), and there were assessments that were missed for reasons unrelated to patient factors (e.g. equipment unavailable) were considered “not applicable” and the total number of possible assessments was modified (15 minus the number of “not applicable” assessments). We refer to the total number of possible assessments for patient i as n_i. If the patient missed the follow-up visit, then the patient missed all n_i of the n_i possible assessments; otherwise, the outcome for the patient is defined as the number of the n_i possible assessments missed, referred to as y_i. The data, y_i and n_i, are available for each patient each follow-up until 24-months or patient death.

At a single follow-up, the outcome, $y_i = 0, 1, 2, \ldots, n_i$, is assumed to follow a Binomial distribution with mean *pi*, the probability of not completing an assessment, assumed to be the same for each of the n_i assessments. In the special case that a single assessment was administered at each follow-up, the outcome distribution reduces to the Bernoulli distribution, i.e. $y_i = 0$ or 1 with mean p_i and number of trials $n_i = 1$. A regression model for the mean p_i, the probability of a missed assessment, can be specified using the generalized linear model theory, (see McCullagh and Nelder pages 101-135; the likelihood function is presented on page 114)[1] which is derived for the general Binomial distribution, for which the Bernoulli distribution is a special case. This generalized linear model theory motivated our choice of the Binomial regression model with a logit link function. Moreover, binomial regression models have been applied in similar settings; for instance, in studies of asthma, authors count the number of days with asthma symptoms over a fixed follow-up period (e.g. two weeks) and model the log odds of a day with asthma symptoms as a function of weight status.[2]

Table S1. Number (percentage) of missed assessments* over 3-, 6-, 12, and 24-month follow-up after ARDS

	**3 Months N=174**	**6 Months N=173**	**12 Months N=156**	**24 Months N=146**	**Total N=649**
EQ-5D	12 (7)	5 (3)	6 (4)	6 (4)	29 (5)
Short-Form 36	16 (9)	6 (4)	8 (5)	6 (4)	36 (6)
Activities of Daily Living	5 (3)	1 (1)	1 (1)	1 (1)	8 (1)
Instrumental Activities of Daily Living	5 (3)	1 (1)	1 (1)	1 (1)	8 (1)
Telephone Interview of Cognition Status	17 (10)	10 (6)	10 (7)	8 (6)	45 (7)
Miscellaneous (Employment, Caregiver, etc.)	5 (3)	1 (1)	2 (1)	1 (1)	9 (1)
Hospital Anxiety and Depression Scale	17 (10)	9 (5)	11 (7)	6 (4)	43 (7)
Impact of Event Scale-Revised	18 (11)	10 (6)	12 (8)	7 (5)	47 (7)
Hearing Handicap Inventory for Adults-Screening	15 (9)	10 (6)	11 (7)	8 (6)	44 (7)
Sydney Swallowing Questionnaire	27 (16)	27 (16)	25 (17)	10 (7)	89 (14)
Anthropometric measures	51 (31)	38 (23)	25 (16)	16 (11)	130 (21)
6-minute Walk Distance	60 (38)	44 (27)	33 (22)	28 (20)	165 (27)
Hand Grip Strength Testing	40 (24)	27 (16)	23 (15)	12 (9)	102 (16)
Manual Muscle Testing	36 (21)	26 (15)	23 (15)	12 (8)	97 (15)
Maximum Inspiratory Pressure	44 (28)	32 (20)	26 (18)	13 (9)	115 (19)

* Excludes assessments not applicable to some participants (e.g. contraindications, comatose/cognitive status, amputated limbs or digits) and those missed for reasons unrelated to participant factors (e.g. staff or equipment unavailable to conduct assessment). Counts for missing assessments are for assessments missing in their entirety. Partially completed assessments were not considered missed.

Table S2. Results of Regression Diagnostics

		6-12-24 month Model[e]			
	3-month Model[d]	Exchangeable correlation[f]	Exchangeable correlation + IES-R- time interaction	Unstructured correlation	AR1 correlation
QICu[a]	Not applicable	61.97	68.47	62.22	62.09
Cooks D (Min, Max)[b]	(0.000, 0.126)	(0.000, 0.055)	(0.000, 0.044)	(0.000, 0.054)	(0.000, 0.051)
VIF (Min, Max)[c]	(1.021, 1.069)	(1.04, 1.50)	(1.02, 2.66)	(1.04, 1.50)	(1.04, 1.50)

Abbreviations: QIC, Quasi-Information Criteria; AR1, Autoregressive 1; VIF, Variance Inflation Factors; IES-R, Impact of Event Scale-Revised

[a] Quasi-information criteria to assess model fit

[b] Cooks D to assess for influential data points

[c] Variance inflation factors to assess for multicollinearity

[d] Diagnostic results for multivariable Binomial logistic regression model in Table 3

[e] Longitudinal Binomial logistic regression models fit with generalized estimating equations

[f] Diagnostic results for multivariable Binomial logistic regression model in Table 4

Figure S1. Mean logit of proportion of missed assessment for variables included in the 3-month multivariable model (see Table 3 of the paper)

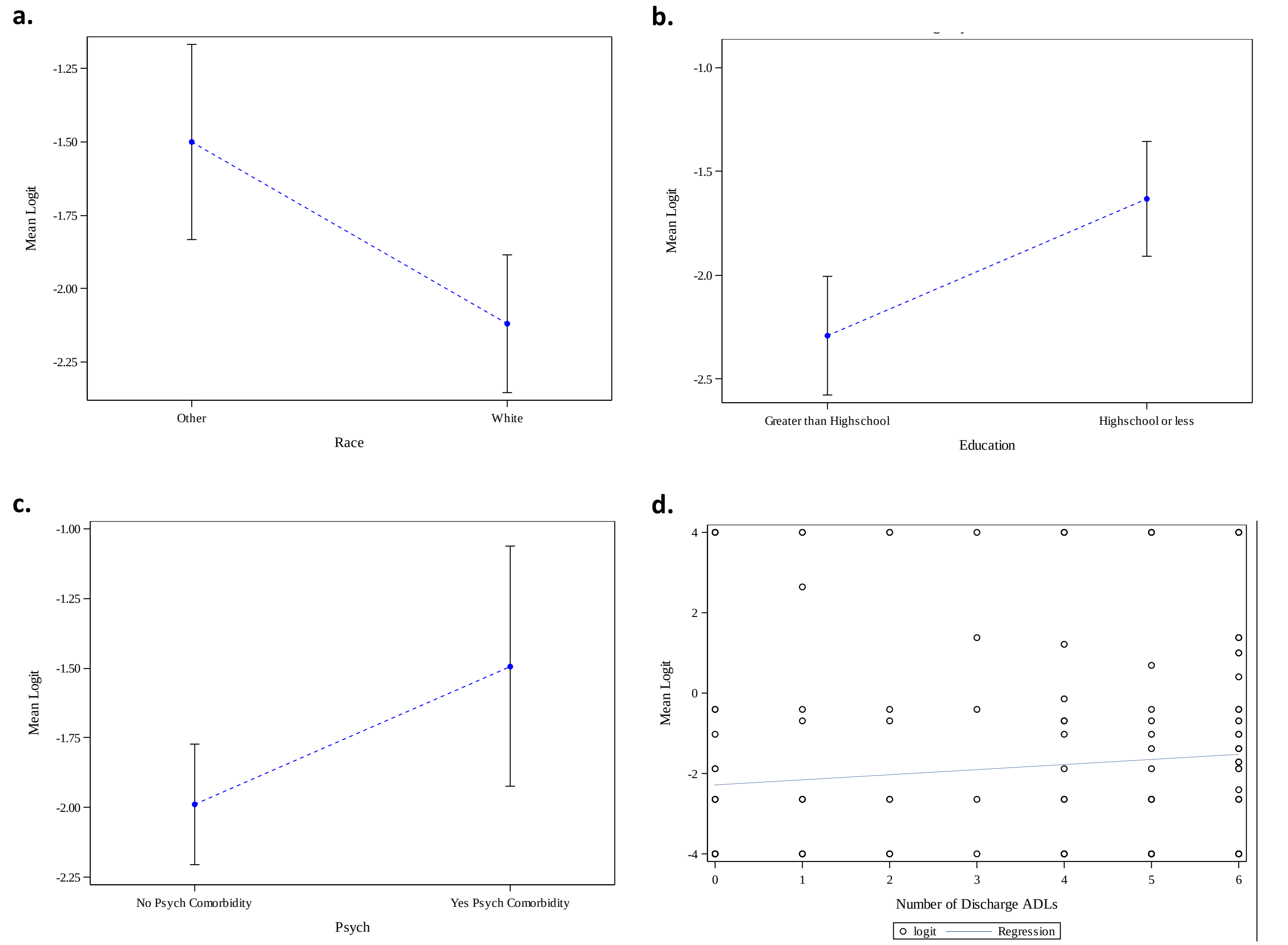

Figures a-c illustrate the mean logit of proportion of missed assessments with corresponding 95% confidence interval (error bars) for each binary predictors. Figure d illustrates regression line of proportion of missed assessments (with scatter plot of raw data) for the continuous predictor 'Number of Discharge ADLs'. Logit of proportion of missed assessments was undefined when probability was 0 or 1; therefore these logits were set to -4 and 4, respectively, for the purpose of these illustrations. The lower the mean logit, the lower the odds of a missed assessment. As mean logit increases, the higher the odds of a missed assessment.

Figure S2. Mean logit of proportion of missed assessment for variables included in the 6-12-24-month multivariable model (see Table 4 of the paper)

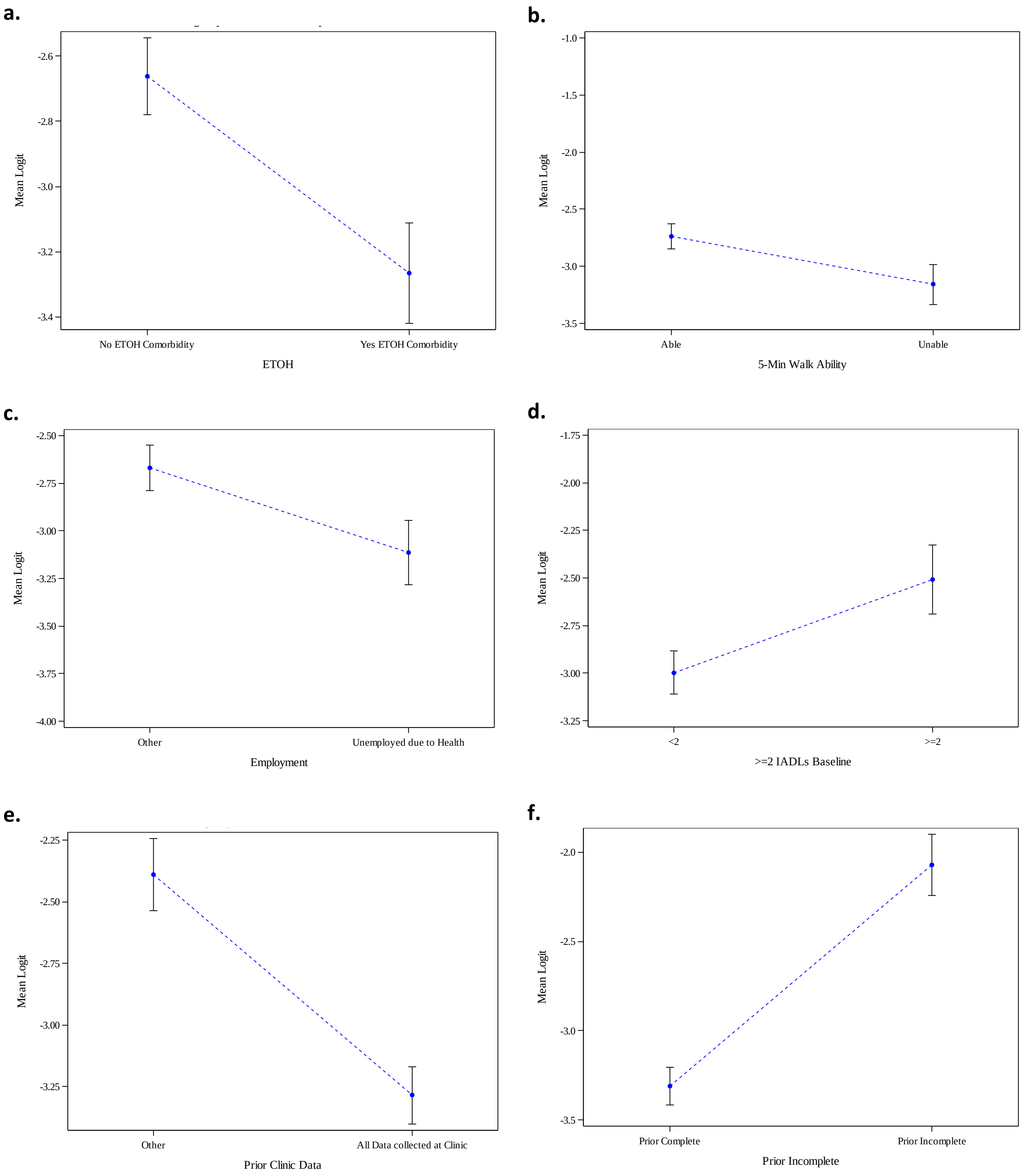

g.

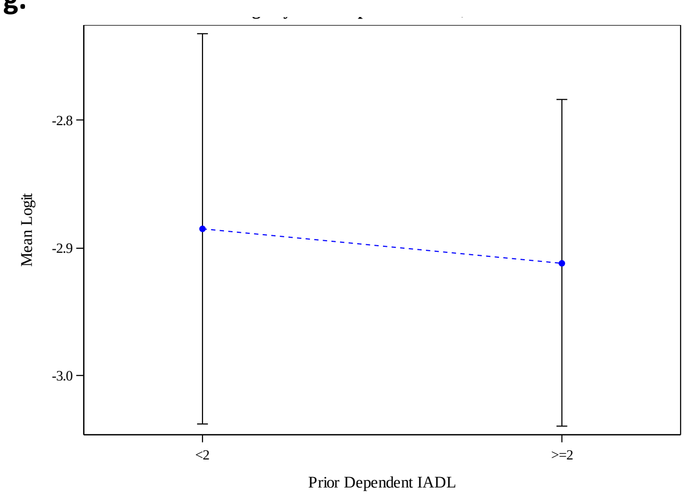

Figures illustrate unadjusted mean logit of proportion of missed assessments with corresponding 95% confidence interval (error bars) for each binary predictors in the 6-12-24 month model. Logit of proportion of missed assessments was undefined when probability was 0 or 1; therefore these logits were set to -4 and 4, respectively, for the purpose of these illustrations. The lower the mean logit, the lower the odds of a missed assessment. As mean logit increases, the higher the odds of a missed assessment.

References

1. McCullagh P, Nelder JA. *Generalized Linear Models, second edition*. Chapman and Hall/CRC. Boca Raton, Florida, 1998.

2. Lu KD, Breysse PN, Diette GB, Curtin-Brosnan JC, Aloe C, Williams DAL, Peng RD, McCormack MC, Matsui EC. Being overweight increases susceptibility to indoor pollutants among urban children with asthma. *J Allergy Clin Immunol*. 2013 Apr; 131(4): 1017 – 1023.
